# Nephrotic Syndrome During Ipilimumab Plus Nivolumab Therapy for Sarcomatoid Malignant Pleural Mesothelioma: A Case Report of Nivolumab Rechallenge Without Recurrence of Nephrotic Syndrome

**DOI:** 10.7759/cureus.115962

**Published:** 2026-09-08

**Authors:** Kazuhiro Shimaya, Jun Ota, Tomoka Yasuda, Makoto Izumi, Shun Endo, Yoshikazu Tsukada

**Affiliations:** 1 Department of Respiratory Medicine, Soka Municipal Hospital, Soka, JPN; 2 Department of Nephrology, Soka Municipal Hospital, Soka, JPN

**Keywords:** ici rechallenge, immune checkpoint inhibitor, ipilimumab, malignant pleural mesothelioma, nephrotic syndrome, nivolumab

## Abstract

Immune checkpoint inhibitor (ICI)-associated nephrotic syndrome is a rare immune-related adverse event. When it occurs during combination ICI therapy, identifying the causative agent and determining an appropriate strategy for subsequent ICI rechallenge can be clinically challenging. A 67-year-old man with unresectable sarcomatoid malignant pleural mesothelioma received combination therapy with ipilimumab and nivolumab, resulting in tumor shrinkage. Approximately four months after treatment initiation, he developed nephrotic syndrome characterized by severe proteinuria, hypoalbuminemia, and bilateral pleural effusions. Both ICIs were discontinued, and prednisolone therapy resulted in improvement of nephrotic syndrome. However, tumor regrowth was observed during corticosteroid tapering, and nivolumab monotherapy was selected for rechallenge. Nephrotic syndrome did not recur after nivolumab rechallenge, which was administered with concurrent prednisolone at a tapering dose. This pattern raises the possibility that ipilimumab alone or the combination of ipilimumab and nivolumab contributed to the development of nephrotic syndrome. This case provides clinical insight into the potential contribution of individual ICIs to ICI-associated nephrotic syndrome and into selective ICI rechallenge as a subsequent treatment strategy.

## Introduction

Immune checkpoint inhibitors (ICIs) are known to cause a wide range of immune-related adverse events (irAEs). Renal toxicity is a relatively uncommon irAE, with acute tubulointerstitial nephritis being the most frequently reported manifestation [1]. In recent years, however, glomerular diseases, including minimal change disease (MCD), membranous nephropathy, IgA nephropathy, and focal segmental glomerulosclerosis, have also been reported [2]. An increasing number of case reports and case series of ICI-associated nephrotic syndrome have been published [3,4].

Combination therapy with anti-cytotoxic T-lymphocyte-associated protein 4 (CTLA-4) and anti-programmed cell death protein 1 (PD-1) antibodies has become widely used in cancer treatment but carries a higher incidence of treatment-related adverse events than monotherapy [5]. ICI-associated glomerular injury with severe proteinuria has been reported after treatment with the anti-CTLA-4 antibody ipilimumab alone and after combination therapy with ipilimumab and the anti-PD-1 antibody nivolumab [3,6-10]. When an irAE develops during combination ICI therapy, determining the contribution of each agent is often difficult, and the role of CTLA-4 inhibition in ICI-associated nephrotic syndrome remains unclear.

Here, we report a case of nephrotic syndrome that developed during ipilimumab plus nivolumab therapy for sarcomatoid malignant pleural mesothelioma. After improvement with corticosteroid therapy, nivolumab monotherapy was reintroduced without recurrence of nephrotic syndrome. Although the clinical course does not establish the causative agent, it raises the possibility that ipilimumab alone or the combination of ipilimumab and nivolumab contributed to the development of nephrotic syndrome.

## Case presentation

A 67-year-old man with a history of type 2 diabetes mellitus presented with right-sided chest pain in March 2025. Chest radiography revealed a pleural-based mass in the lateral right lower lung field and a right pleural effusion (Figure 1A). Chest computed tomography (CT) demonstrated three mass lesions adjacent to the right pleura and a right pleural effusion (Figures 1B-1D). One of the lesions was associated with a rib fracture caused by chest wall invasion (Figure 1E).

**Figure 1:**
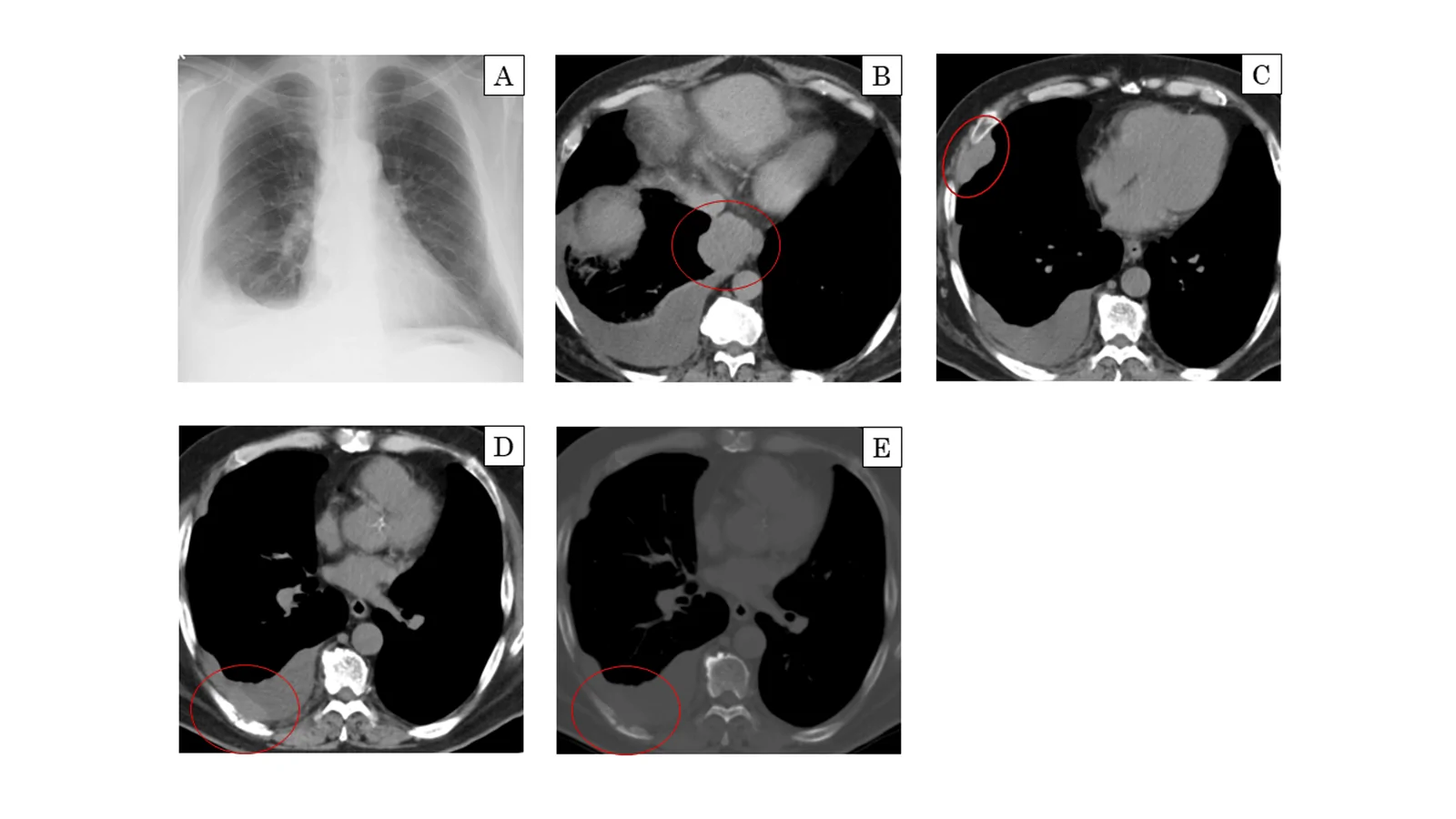
Chest imaging findings before treatment initiation. (A) Chest radiography shows a pleural-based mass in the lateral right lower lung field and a right pleural effusion. (B-D) Chest CT shows three mass lesions adjacent to the right pleura and a right pleural effusion. (E) One of the lesions is associated with a rib fracture caused by chest wall invasion.

Laboratory tests showed a serum albumin level of 3.1 g/dL (reference range: 3.9-4.9 g/dL), urinary protein of 2+ (reference range: negative), and a serum creatinine level of 0.99 mg/dL (reference range: 0.61-1.04 mg/dL). First-line combination therapy with ipilimumab (1 mg/kg every six weeks) and nivolumab (360 mg every three weeks) was initiated (Day 0).

On Day 107, the scheduled dose of nivolumab was withheld because the serum albumin level had declined to 1.7 g/dL, raising clinical suspicion of nephrotic syndrome. The patient had received three doses of ipilimumab and five doses of nivolumab and was subsequently referred to the nephrology department. On Day 134, approximately four months after treatment initiation, CT demonstrated tumor shrinkage; however, the right pleural effusion had increased, and a new left pleural effusion had developed (Figure 2A). Bilateral lower-extremity edema was also present. The serum albumin level had further decreased to 1.6 g/dL, and urinalysis revealed 4+ proteinuria with a spot urine protein-to-creatinine ratio of 21.5 g/gCr (reference range: <0.15 g/gCr). These findings were consistent with nephrotic syndrome. Serum creatinine was 0.97 mg/dL, with no apparent deterioration in renal function.

**Figure 2:**
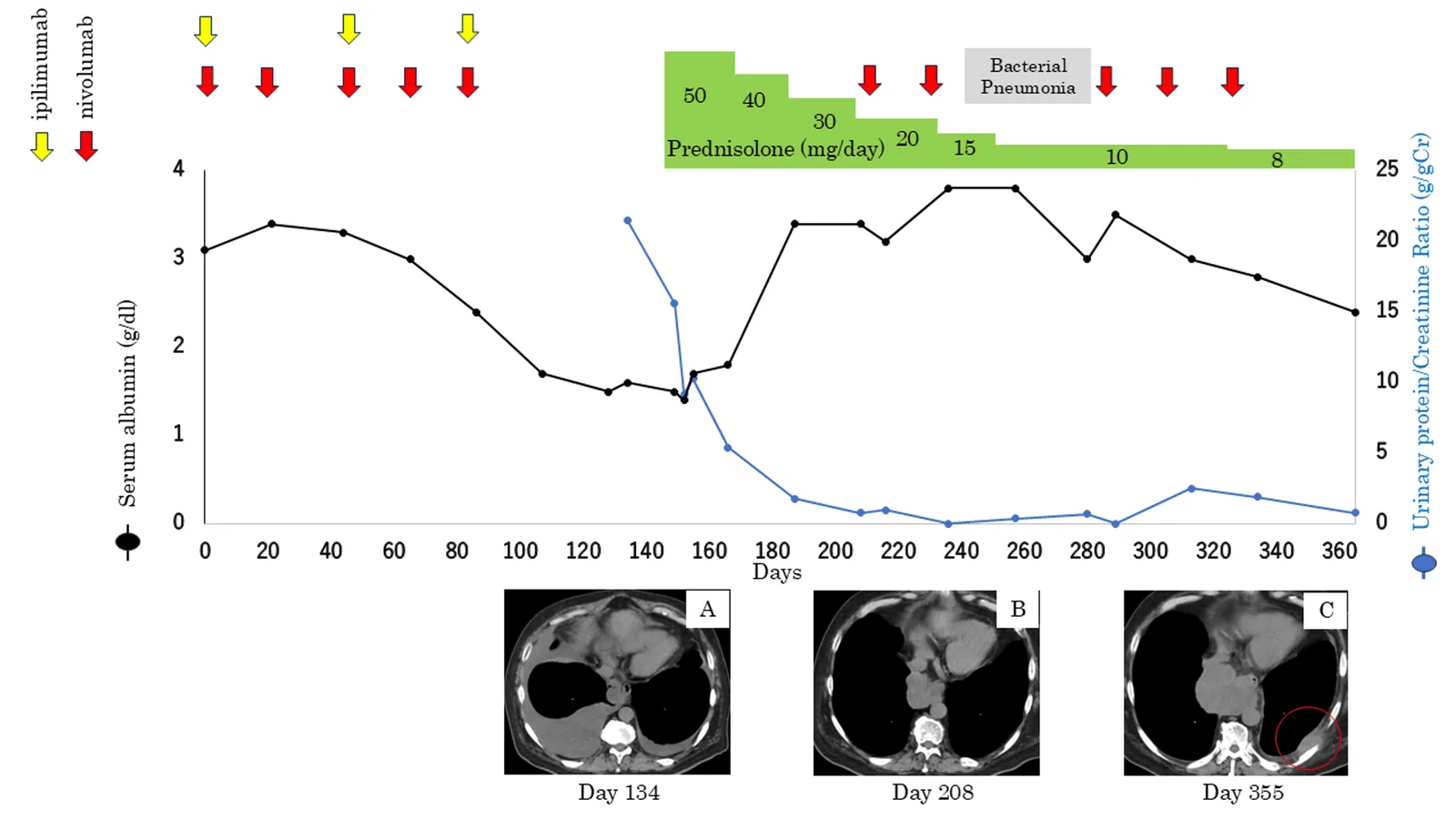
Clinical course and chest CT findings. The clinical course depicts the timing of ipilimumab and nivolumab administration, the prednisolone dose, the urine protein-to-creatinine ratio, and serum albumin levels. (A) Day 134: The tumors had decreased in size; however, the right pleural effusion had increased, and a new left pleural effusion had developed. (B) Day 208: The bilateral pleural effusions had resolved, but tumor regrowth was observed. (C) Day 355: Further tumor progression and left pleural dissemination were observed.

Evaluation for secondary causes of nephrotic syndrome showed negative results for antinuclear antibodies and antineutrophil cytoplasmic antibodies, normal complement levels, and no evidence of hepatitis B or C virus infection. There was no history of medications that could exacerbate proteinuria, such as nonsteroidal anti-inflammatory drugs. Mild proteinuria was present before ICI initiation, and an underlying chronic glomerular disease, including diabetic nephropathy, could not be excluded. However, the rapid development of marked proteinuria and hypoalbuminemia after ICI initiation raised suspicion of ICI-associated nephrotic syndrome.

Both ipilimumab and nivolumab were discontinued. The diagnostic value and potential complications of renal biopsy, including bleeding, were discussed with the patient. However, because of concerns related to his comorbidities, including diabetes mellitus, and because consent for the procedure could not be obtained, renal biopsy was not performed. Prednisolone (1 mg/kg/day; 50 mg/day) was initiated on Day 149. Proteinuria and hypoalbuminemia subsequently improved rapidly. By the time prednisolone had been tapered to 20 mg/day, the serum albumin level had increased to 3.4 g/dL, urinary protein had decreased to 2+, and the urine protein-to-creatinine ratio had decreased to 0.8 g/gCr.

Meanwhile, chest CT on Day 208 showed resolution of the pleural effusions but tumor regrowth (Figure 2B). Because the initial combination of ipilimumab and nivolumab had achieved tumor shrinkage and treatment options for malignant pleural mesothelioma were limited, ICI rechallenge was considered. However, because nephrotic syndrome had developed during combination therapy, the specific causative agent could not be identified. Given concern regarding recurrence of the irAE with reintroduction of combination therapy, ipilimumab was not reintroduced, and nivolumab monotherapy was restarted on Day 216 while prednisolone was continued at 20 mg/day.

After two doses of nivolumab, the patient developed bacterial pneumonia requiring antibiotic therapy, and nivolumab was temporarily discontinued. Nivolumab was resumed after resolution of the pneumonia. During subsequent treatment, gradual tumor progression was observed, but nephrotic syndrome did not recur. Chest CT on Day 355 demonstrated further tumor progression and left pleural dissemination (Figure 2C); treatment was subsequently switched to pemetrexed.

## Discussion

The notable clinical feature of this case was the development of nephrotic syndrome during combination therapy with ipilimumab and nivolumab, with subsequent improvement after discontinuation of both agents and corticosteroid treatment, without recurrence after rechallenge with nivolumab monotherapy. In addition to the rarity of ICI-associated nephrotic syndrome, this case provides a clinically informative course for considering the potential contribution of individual ICIs to nephrotic syndrome occurring during combination therapy and for selecting an ICI regimen for subsequent rechallenge.

Previous reports of glomerular injury associated with ipilimumab alone or ipilimumab plus nivolumab are summarized in Table 1.

**Table 1:** Reported cases of nephrotic-range proteinuria or nephrotic syndrome following ipilimumab-containing therapy, including the present case. Alb, albumin; Cr, creatinine; ICI, immune checkpoint inhibitor; MCD, minimal change disease; NR, not reported; NSCLC, non-small-cell lung cancer.

Reference	Age, Sex	Malignancy	ICI Regimen	Onset after Ipilimumab	Renal Pathology	Renal Findings at Onset	Treatment	ICI Rechallenge and Outcome
Fadel et al. [6]	64, M	Melanoma	Ipilimumab	After two doses	Lupus nephritis	Cr 1.0 mg/dL; Alb 2.45 g/dL; proteinuria 7.5 g/day	Ipilimumab discontinued; prednisone 1 mg/kg/day started later (after renal vein thrombosis)	No rechallenge reported
Kidd & Gizaw [7]	55, M	Melanoma	Ipilimumab	NR (not specified in report)	Minimal change disease with interstitial nephritis	Cr 2.97 mg/dL (peak 5.2 mg/dL); Alb 2.2 g/dL; proteinuria 9 g	Ipilimumab discontinued; high-dose steroids (2 mg/kg)	No rechallenge reported
Kitchlu et al. [8]	45, M	Melanoma	Ipilimumab	4th dose (~18 mo into treatment course)	Minimal change disease	Cr 0.8 mg/dL; Alb 2.6 g/dL; proteinuria 9.5 g/day	Ipilimumab discontinued; prednisone 1 mg/kg/day	Ipilimumab rechallenge caused recurrent nephrotic-range proteinuria
Mamlouk et al. [9]	69, M	Melanoma	Ipilimumab + nivolumab	After 2 cycles (~6 wk)	IgA nephropathy with mild acute tubulointerstitial nephritis	Cr 2.4 mg/dL; proteinuria 7.7 g/gCr; Alb NR	Both ICIs discontinued; corticosteroids (0.5 mg/kg/day)	No ICI rechallenge reported; causative contribution of each ICI could not be distinguished
Dougherty et al. [10]	70, F	Stage IV NSCLC	Ipilimumab	4 wk after first ipilimumab cycle	IgA nephropathy with crescents and acute tubular injury	Cr 2.5 mg/dL; 3+ proteinuria; Alb NR	Methylprednisolone 1 mg/kg IV twice daily; AKI progressed despite treatment; hemodialysis required	No rechallenge; temporal association led the authors to attribute the renal irAE to ipilimumab
Saiki et al. [3]	75, M	Malignant pleural mesothelioma	Ipilimumab + nivolumab	13 d after first combination treatment	Minimal change disease with acute interstitial nephritis	Cr 1.87 mg/dL; Alb 2.0 g/dL; proteinuria 19.85 g/day	Both ICIs discontinued; prednisone 1 mg/kg/day; hemodialysis required (35 days)	No ICI rechallenge; causative contribution of each ICI could not be distinguished
Present case	67, M	Sarcomatoid malignant pleural mesothelioma	Ipilimumab + nivolumab	~4 mo after treatment initiation (after 3 ipilimumab doses)	Not available (kidney biopsy not performed)	Cr 0.97 mg/dL; Alb 1.6 g/dL; proteinuria 21.5 g/gCr	Both ICIs discontinued; prednisolone 1 mg/kg/day	Nivolumab monotherapy rechallenge without recurrence of nephrotic syndrome; possible ipilimumab contribution or a combination-specific effect

The report by Kitchlu et al. is particularly informative when considering the association between ipilimumab and nephrotic syndrome [8]. In that report, nephrotic syndrome developed during ipilimumab monotherapy, and renal biopsy demonstrated MCD. Proteinuria improved after discontinuation of ipilimumab and corticosteroid therapy but recurred to the nephrotic range after ipilimumab was reintroduced, suggesting a potential association between ipilimumab and nephrotic syndrome. More recently, CTLA-4 blockade has been reported to impair peripheral B-cell tolerance and promote the emergence of autoreactive B cells, in addition to enhancing T-cell responses [11]. Although this study did not directly investigate glomerular injury, its findings are of interest when considering a potential immunological mechanism through which ipilimumab could contribute to the development of glomerular disease.

Regarding the association between ipilimumab plus nivolumab combination therapy and glomerular injury, an analysis of patients with non-small cell lung cancer using the U.S. Food and Drug Administration Adverse Event Reporting System demonstrated a disproportionality signal for glomerular disorders, including nephrotic syndrome, with ipilimumab plus nivolumab combination therapy compared with nivolumab monotherapy [12]. These findings suggest a potential association between adding ipilimumab to nivolumab and glomerular injury, including nephrotic syndrome; however, because the analysis was based on a spontaneous adverse-event reporting database, it cannot establish causality or determine the contribution of each agent.

In the present case, ipilimumab and nivolumab were administered concurrently at the onset of nephrotic syndrome; therefore, the contribution of each agent could not be determined from the clinical course alone. Because tumor regrowth occurred during interruption of ICI therapy, ICI rechallenge was considered. Given concern regarding recurrence of the irAE with reintroduction of combination therapy and the limited treatment options available for malignant pleural mesothelioma, ipilimumab was not reintroduced, and nivolumab monotherapy was selected; no recurrence of nephrotic syndrome was subsequently observed.

However, the absence of recurrence after nivolumab rechallenge does not exclude a contribution from nivolumab or establish ipilimumab as the causative agent. irAEs do not invariably recur after re-exposure to an ICI. Moreover, the patient remained on prednisolone at 20 mg/day when nivolumab was reintroduced, and its immunosuppressive effect may have prevented recurrence. Nephrotic syndrome may also have resulted from the combined effects of CTLA-4 and PD-1 blockade rather than either agent individually. Therefore, although the clinical course cannot establish ipilimumab as the cause of nephrotic syndrome, it provides clinical insight into its potential contribution. Further accumulation of similar cases, with detailed evaluation of the ICI regimen, use of combination therapy, renal pathological findings, and clinical course after rechallenge, will be important for clarifying the roles of individual ICIs in ICI-associated glomerular injury.

Various glomerular lesions, including MCD, membranous nephropathy, focal segmental glomerulosclerosis, and IgA nephropathy, have been reported in patients with ICI-associated nephrotic syndrome [3]. In the present case, ICI-associated glomerular injury was clinically suspected because of the acute onset of severe proteinuria and hypoalbuminemia after ICI administration and the favorable response to corticosteroid therapy. The selectivity index was 0.20, which did not meet the criterion for highly selective proteinuria. Given the patient's history of type 2 diabetes mellitus and mild proteinuria before ICI initiation, pre-existing glomerular injury, such as diabetic nephropathy, may have influenced this finding. Nevertheless, the rapid development of severe proteinuria and hypoalbuminemia after ICI initiation, together with the favorable response to corticosteroid therapy, was difficult to explain by diabetic nephropathy alone and supported the possibility of ICI-associated glomerular injury. The inability to perform renal biopsy and determine the histopathological subtype, together with the presence of proteinuria before ICI initiation, are important limitations of this case and preclude a definitive causal relationship between ICI therapy and nephrotic syndrome.

## Conclusions

We report a case of nephrotic syndrome that developed during combination therapy with ipilimumab plus nivolumab, improved after discontinuation of both agents and corticosteroid treatment, and did not recur after rechallenge with nivolumab monotherapy, although concurrent corticosteroid therapy during the rechallenge period may have contributed to the absence of recurrence. This case illustrates a clinical approach to rechallenging with an individual ICI following an irAE of uncertain causative attribution during combination therapy. Evidence regarding rechallenge strategies after ICI-associated nephrotic syndrome remains limited, and accumulation of similar cases may help inform safer treatment strategies.
